# The cell pushes back: The Arp2/3 complex is a key orchestrator of cellular responses to environmental forces

**DOI:** 10.1016/j.ceb.2020.08.012

**Published:** 2021-02

**Authors:** Vassilis Papalazarou, Laura M. Machesky

**Affiliations:** 1 CRUK Beatson Institute for Cancer Research, Garscube Estate, Switchback Road, Glasgow, G61 1BD, UK; 2 Institute of Cancer Sciences, Garscube Estate, University of Glasgow, Glasgow, G61 1BD, UK

**Keywords:** Mechanosensing, Actin, Arp2/3 complex, Cell migration, Metabolism, Endocytosis

## Abstract

The Arp2/3 complex orchestrates the formation of branched actin networks at the interface between the cytoplasm and membranes. Although it is widely appreciated that these networks are useful for scaffolding, creating pushing forces and delineating zones at the membrane interface, it has only recently come to light that branched actin networks are mechanosensitive, giving them special properties. Here, we discuss recent advances in our understanding of how Arp2/3-generated actin networks respond to load forces and thus allow cells to create pushing forces in responsive and tuneable ways to effect cellular processes such as migration, invasion, phagocytosis, adhesion and even nuclear and DNA damage repair.

## Introduction

The Arp2/3 complex represents a unique molecular machine that enables the formation of branched actin filaments at the surfaces of lipid membranes. The Arp2/3 complex was first described in *Acanthamoeba* and mammalian cells as a 7-protein complex that nucleates actin filaments and localises to dynamic actin structures [1,2]. Uniquely among actin-nucleating proteins, it engages with the side of an actin filament and nucleates a branch at a 70° angle, with the new barbed (fast-growing) ends pointing toward the membrane surface. Actin polymerisation by Arp2/3 is referred to as dendritic nucleation [3], and the assembly of these networks generates pushing forces against the membrane surface. Thus, these actin networks resist membrane tension or forces exerted by other objects. The 7 subunits of the Arp2/3 complex are Arp2 and Arp3 (actin-related protein-2 and -3), Arpc1, Arpc2, Arpc3, Arpc4 and Arpc5 which form a tight complex. Mammals have isoforms of many of these subunits [4,5], some of which have differing nucleation capabilities [4], but the individual functions of each isoform are not yet well understood.

The Arp2/3 complex is an inefficient nucleator of actin by itself and requires a nucleation promoting factor to activate it. The most well understood family of nucleation promoting factors is the WASP family, which consists of WASP/N-WASP, Scar/WAVE1-3, WASH, JMY and WHAMM proteins. WASP/N-WASP and Scar/WAVE proteins act at the plasma membrane, with WASP and N-WASP also triggering branched actin formation during membrane trafficking. WASH, JMY and WHAMM all act on internal membranes including endocytic vesicles, the Golgi complex and autophagosomes. Nucleation promoting factors help to specify not only the activity status of the Arp2/3 complex, but also the location where it will be activated. The WASP family of nucleation promoting factors have been extensively reviewed elsewhere [6,7] and are thus only briefly described here.

## Arp2/3 at the leading edge of the cell

The best understood function of the Arp2/3 complex is to nucleate actin underneath the plasma membrane to effect protrusion of the cell edge. These protrusions are known as lamellipodia and form membrane sheets that can either remain adherent to the substratum or ruffle back in a wave form toward the cell body (see Box 1 for terminology). Cells moving across a rigid flat substratum use lamellipodia for haptotaxis [8]. Cells in 3D environments rather use Arp2/3 to generate force that creates invasive finger-like protruding structures, such as invadopodia, found in cancer cells, or podosomes in the case of some haematopoietic cells, endothelial cells and the *Caenorhabditis elegans* anchor cell that are characterised by a highly dynamic actin-comet-like appearance [9,10]. Arp2/3-dependent branched actin networks could provide mechanosensitive properties to these structures [11]. Cells also use the Arp2/3 complex at the plasma membrane to generate macropinocytic and phagocytic cups, structures used to engulf fluids or large particles, respectively [12].

Box 1 **– Terminology**

**Actin-nucleating proteins**

Protein complexes that catalyse actin polymerisation serving as initiation sites for new actin filaments.

**Dendritic nucleation**

The Arp2/3 complex nucleates a new (‘daughter’) filament from the side of the pre-existing (‘mother’) filament. Monomers add to barbed ends of both filaments, until capping by a barbed-end capping protein. This activity allows formation of branched actin networks.

**Nucleation promoting factor**

Proteins that direct nucleation of actin filaments by recruiting actin monomers to pre-existing actin filaments and directly binding to and activating the Arp2/3 complex.

**Lamellipodia**

Thin, sheet-like membrane protrusions typically found in the leading edge of motile cells in 2D.

**Haptotaxis**

Directional motility of cells in response to a gradient of cellular adhesion sites or surface-bound proteins or chemoattractants.

**Invadopodia**

Dynamic actin-rich structures of the plasma membrane that are typically found in cancer cells and are involved with ECM degradation during invasion.

**Podosomes**

Dynamic actin-rich and ECM-degrading cellular protrusions typically found in endothelial, immune and other physiological cell types.

**Capping proteins**

Proteins that regulate actin polymerisation by binding to barbed ends of growing actin filaments and blocking further exchange of subunits at these ends.

**Pseudopodia**

Finger-like projections of the cytoplasm of eukaryotic cells commonly observed in cells invading 3D or fibrillar ECM.

**Focal adhesions**

Adhesive contacts between cells and the ECM mediated through the interaction of transmembrane proteins such as integrins to ECM ligand sites as well as intracellular protein complexes that are connected to actin filaments.

**Adherens junctions**

Cell-to-cell junction whose cytoplasmic face is directly linked to actin filaments. They are typically composed of cadherin receptors and cytoplasmic adaptor proteins (catenins).

Alt-text: Box 1

Cells actively adapt to their environment, including responding to physical forces imposed by the external world. It has recently emerged that branched actin networks assembled under force respond to the load by increasing the number of actin filament barbed ends [13]. Branched actin networks behave very differently to randomly crosslinked networks, which also respond to load forces [14,15], thus giving the actin generated by Arp2/3 special properties to carry out specific cellular functions. Not only are the different operators in branched actin networks (capping proteins, Arp2/3 and actin filaments) differently force sensitive, but the whole geometry of these networks changes under mechanical load in a responsive way. Mueller et al. [16] found that by applying suction to the rear of a migrating keratocyte, they could increase membrane tension at the front and thus induce a mechanoadaptation of the branched actin network in the leading edge. They propose that filament-barbed ends underneath the plasma membrane are protected from capping, promoting increased filament density locally. Specifically, at steady state, filaments in migrating cells display the canonical dendritic geometry, defined by Arp2/3-generated 70° angle dendritic branching. Increased tension induced generation of dense filament networks displaying a broad range of angles. In contrary, decreased tension was associated with sparse filament distribution with filaments growing perpendicularly to the plasma membrane. Using a combination of quantitative light and electron microscopy as well as stochastic modelling, they described how the filament network structure geometrically adapts to counter forces to explain how protrusive force is generated upon mechanical load [16]. It would be interesting to know whether close proximity to certain plasma membrane lipids, for example, phosphoatidylinositol 4,5 bisphosphate, might also selectively remove caps from the barbed ends, as was previously shown to occur *in vitro* [17]. It is clear that branched actin networks can adapt to mechanical forces in various ways and that they retain some memory of the force load under which they were first generated [13]. Branched actin networks also relax more slowly and undergo rare convulsive, strain-release movements compared with unbranched networks [18]. Not only the formation, but also dissociation of actin filament branches can depend on Arp2/3 activity. Two distinct mechanical states of the Arp2/3 complex have been described with different stability to force, with a released phosphate state to favour debranching upon force [19]. In addition to allowing the cell to respond with variable force in lamellipodia protrusions, these features give branched actin networks a versatility allowing unique roles in cells and tissues.

Cells migrating in 3D or fibrillar environments show elongated pseudopodia, and their leading edge engages with a network of microtubules as well as Arp2/3-driven actin assembly. A recent study revealed that Arp2/3-generated branched actin could influence microtubule stability through its effect on HDAC-6–mediated acetylation of tubulin [20]. HDAC6 can be sequestered by cortactin, which is abundant in branched actin networks, thus decreasing the de-acetylation of tubulin and leading to an increase in acetylated, stable tubulin [20]. Interestingly, this affects the distribution of mitochondria, as the acetylation status directs whether kinesins, such as Kif5b (anterograde transport), or dyneins (retrograde transport) would drive mitochondrial transport. This gives an intriguing explanation for how mitochondria might traffic along pseudopodia via dynamic Arp2/3-mediated actin assembly.

## Arp2/3 in clathrin-mediated endocytosis

**Figure 1:**
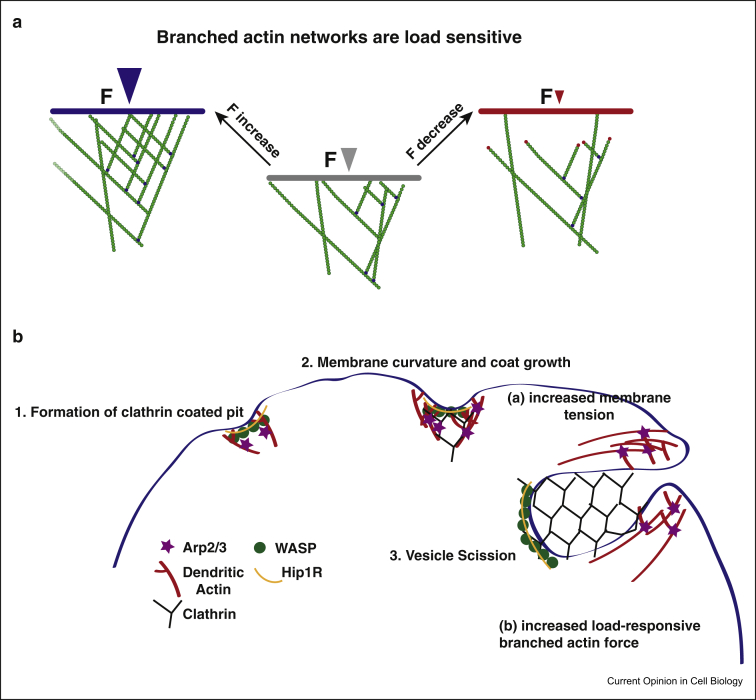
**Branched actin networks are load-sensitive and direct membrane dynamics. (A)** Branched actin networks are load-sensitive. Scheme shows actin filaments growing at steady state with intermediate force load (middle, grey arrow). An increase in load (left, blue arrow) increases filament density, whereas after a decrease in load (right, red arrow) density decreases and branched filaments proximal to the membrane are preferentially capped (red). The Arp2/3 complex is highlighted in blue. F; Force. Figure adapted from the study by Mueller et al. [16]. **(B)** Spatially constrained branched actin networks enable endocytic uptake of clathrin-coated pits. Generation of force by the actin assembly shapes the dynamics of plasma membrane. This is especially evident by the manifestations of the actin cytoskeleton during clathrin-mediated endocytosis (CME). Actin is organised as a radial branched array with growing ends facing the base of the pit. During endocytosis, long actin filaments bend between attachment sites in the coat and the base of the endocytic vesicle, and therefore endocytic internalisation depends on elastic energy stored in bent filaments. This is based on the neck of the endocytic vesicle being a flexible spring and generating tension between the plasma membrane and the nascent vesicle. It has been shown that a band of the actin capturing molecule Hip1R near the internal distal end of the vesicle could capture actin filaments and allow load-responsive polymerisation of Arp2/3-induced dendritic networks to oppose the membrane tension forces. Increased membrane tension directs more growing filaments toward the base of the pit increasing actin nucleation and bending locally and therefore increased force production derived from branched actin. Spatially constrained actin filament assembly therefore enables endocytosis [21].

In addition to its role in lamellipodia, Arp2/3 plays a crucial and conserved role in clathrin-mediated endocytosis (CME). Formation of endocytic vesicles requires dynamic changes in the plasma membrane. Through a stepwise process of pulling, sculpting and pushing, plasma membrane mechanics allow endocytic vesicle invagination, maturation and ultimately scission. Thus, this process involves increased membrane tension over time and spatially, and these forces are mediated through branched actin networks. Akamatsu et al. [21] and Sun et al [22] recently modelled how Arp2/3-generated actin networks assist CME events, using agent-based modelling and quantitative experimental studies. They found that endocytic actin networks adapted to the changing landscape of membrane tension during the various stages of deformation and endocytic uptake of clathrin-coated endocytic vesicles (Figure 1). Their model elucidated how load adaptation of actin branching could allow cells to adjust to higher or lower membrane tension situations. It can also possibly explain differences observed between yeast, which requires actin for CME because of high turgor pressure, and other cell types with lower turgor pressure [22]. Clathrin- and dynamin-independent endocytosis was reported to also depend on Arp2/3-mediated actin dynamics for vesicle formation [23]. Arp2/3-dependent branched actin polymerisation is also important for generation of actin foci that are interspersed with linear filaments and myosin-IIa in B cells. These Arp2/3-dependent structures drive antigen internalisation implicating the mechanical properties of branched actin in immunity [24]. Specifically, both the Arp2/3 complex and formins appeared to be needed for antigen uptake by B cells by the creation of a focal filament network complex that through local bursts of actin polymerisation generates an inward force driving antigen internalisation. These actin polymerisation-dependent processes are highly important for naïve or memory B cells where antigen extraction is directly followed by endocytosis but could also drive other, nonendocytosis coupled antigen extraction and transport mechanisms, such as the ones that exist in germinal centre B cells, suggesting Arp2/3-mediated dendritic branching as a global player for antigen extraction from antigen presenting cells [24].

## Arp2/3 at the cell–ECM interface

Cell surface receptors such as integrins are the main mediators of biological and mechanical signals derived from the surrounding microenvironment. Just underneath the plasma membrane, focal adhesions are both signalling and mechanical hubs that directly connect the ECM–integrin interface to the cytoskeleton. Leading edge protrusions appear to depend on integrin activation and signalling components of focal complexes, including FAK or paxillin [25,26]. The Arp2/3 complex has been directly associated with focal adhesion proteins including vinculin and FAK, promoting leading edge formation and cell migration [27, 28, 29]. It is currently thought that the Arp2/3 complex is recruited through Rac1 or phosphatidylinositol-4,5-biphosphate activity to sites of integrin clustering. There it can associate directly with components of focal complexes, such as vinculin, connecting therefore actin polymerisation, membrane protrusion and cell motility to substrate adhesion. Direct binding of the Arp2/3 complex to FAK couples the lamellipodial actin cytoskeleton to focal adhesions and is required for haptosensing [30]. Arp2/3-dependent actin polymerisation induced by WASH is also necessary for recycling of integrins to the plasma membrane [31]. A requirement for the Arp2/3 complex activity in integrin-dependent processes has also been demonstrated in macrophages, where lack of Arp2/3 impaired phagocytosis and haptotaxis, processes exerted by integrin-dependent actin assembly [32]. Thus, the roles of Arp2/3 upon variable extracellular matrix (ECM) environments is worthy of further investigation.

## Arp2/3 at cell–cell junctions

Cells organised in tissues respond to external forces collectively, as a coherent unit. This is usually mediated through adherens junctions that transmit forces and other signals across larger fields. Adherens junctions mediate tissue integrity and give epithelial and endothelial tissues mechanical strength and resilience. While branched actin structures have been described in these junctions before, two recent studies highlight the role of the Arp2/3 complex in generating mechanoresponsive branched actin networks that provide pushing force in response to pulling by myosin-II generated tension inside the cell and by external pressures [33,34]. Epithelial cells are constantly using Arp2/3-generated protrusive fingers or actin microspikes to probe for weaknesses at cell–cell junctions and initiate their repair [34]. These structures depend on the Ena/VASP family member Ena Vasp like (EVL) proteins and the collapsin response mediator protein 1 to elongate the actin filaments through a branching process that is initiated by the Arp2/3 complex. These protrusions seem to sense adherens junctions' defects. Specifically, at places where E-cadherin–mediated contacts have weakened, perhaps because of myosin-II contractile forces pulling the cells apart, they act to repair such weak connections by effecting a protrusion [34]. In a similar study, but using platinum replica electron microscopy to visualise actin and myosin networks at cell–cell junctions in endothelial cells, Efimova and Svitkina [33] found that Arp2/3 is highly concentrated just underneath the plasma membrane in neighbouring endothelial cells and colocalises with VE-cadherin. Beneath the Arp2/3 complex and deeper in the cell, they observe actin–myosin cables, likely responsible for this pulling force observed by Efimova and Svitkina [33] and Li et al. [34]. Overall, Efimova and Svitkina [33] reached a similar conclusion that the zone of Arp2/3 right under the plasma membrane was mechanoresponsive and generated a branched actin-based protrusive force to push the junctions together and keep them robust against external and cell–cell forces [33]. However, the role of the Arp2/3 complex in cell–cell junctions *in vivo* is still unknown. Recent evidence suggested that loss of the ArpC4 subunit in skin keratinocytes results in a psoriasis-like disease in mice [35], and Arpc3 deletion in skin keratinocytes also causes epidermal tight junction and barrier permeability defects [36]. Further studies visualising the role of branched actin in epithelia and endothelial tissues *in vivo* might reveal the contexts where Arp2/3 mechanosensitive pushing forces are important for tissue maintenance and homeostasis.

## Arp2/3 in and around the nucleus

Branched actin networks have also recently been implicated in providing strength and support for cells under stress due to migration in confined spaces. Cell migration in the interstitial spaces of the body can involve squeezing through tight spaces, experiencing high levels of pressure or confinement. These stresses can damage the cell membrane and the nucleus and even cause DNA damage [37,38]. Two studies published in 2019 implicate Arp2/3 in DNA damage repair in the nucleus [39,40]. Caridi et al. [39] showed that Arp2/3 nucleated branched actin networks at repair sites in the nuclear periphery and DNA double-strand break sites were relocalised along these actin networks through myosin activity. Schrank et al. [40], likewise, found that Arp2/3 was recruited to sites where damaged chromatin is being repaired as well as for the movement of repair sites to clusters that seem to specialise in DNA double-strand breaks that are repaired by homology recombination.

Another case where nuclear integrity needs to be compromised is during nuclear envelope breakdown before cell division. In some mammalian cells, microtubules tear the nuclear membrane using force generated by the motor dynein, but a recent study in starfish oocytes showed that the nuclear membrane is ruptured by actin polymerisation driven by the Arp2/3 complex at the surface of the nuclear envelope [41]. Dendritic actin networks form between the lamina and the nuclear envelope, separating them and causing fragmentation of the envelope [41]. Not only nuclear envelope breakdown, but also regulation of symmetry during cell division can be regulated by Arp2/3-generated actin networks. A recent *in vitro* model shows that Arp2/3 and myosin contractile activity form spontaneous contractile waves in metaphase Xenopus egg extracts observed in cell-sized confinement chambers with natural phospholipids [42]. Tissue expansion during embryogenesis requires both symmetrical and asymmetrical divisions, and it seems Arp2/3 merits further investigation as an orchestrator of both mechanical separation of components such as the nuclear envelope and of symmetry and nuclear positioning.

## Connections between the mechanosensitive Arp2/3 actin network and energy flux

Generation of branched actin networks in invadopodia or pseudopods creates a pushing force that helps cells to breach basement membranes and invade through the ECM. Such protrusions recruit mitochondria in close proximity to the dynamic branched actin networks. It is becoming clear that the cell regulates connections between dynamic actin turnover and production of ATP locally to power cytoskeletal dynamics. We and others observed links between invasive behaviour and mitochondrial recruitment to sites where force generation by branched actin are driving invasive protrusion [43,44]. The *C. elegans* anchor cell provides an elegant physiological model of normal developmental invasion during vulval development. Kelley et al. [43] found that although membrane metalloproteinases help to break down the basement membrane to effect invasion, their genetic deletion could be compensated for by excessive branched actin production and accompanying recruitment of mitochondria locally during the invasion. Similarly, we recently found that pancreatic cancer cells are exquisitely mechanosensitive and upregulate their ATP production, mitochondrial respiration and ATP recycling machinery on the ECM resembling the stiff, fibrotic tumour matrix [44]. It has also been shown recently that degradation of phosphofructokinase, an enzyme that catalyses the first rate-limiting step of glycolysis, can be induced by disassembly of stress fibres [45]. Thus, actin networks, including both branched actin and contractile bundles, emerge as important mediators of cell metabolism.

## Outlook

**Figure 2:**
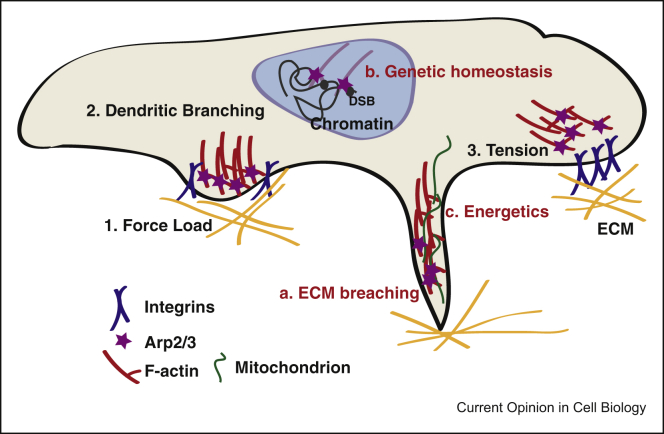
**Arp2/3-dependent actin branching at the roots of the responses to environmental forces.** There is a bidirectional mechanical communication between cells and their environment. Among the main contributors to cell responses against increased force load are (1) branched actin networks (2) which are generated through Arp2/3-mediated dendritic nucleation. Branched actin networks are key in controlling the tension homeostasis (3) at the cytoskeleton–plasma membrane–ECM axis. Dynamics of branched actin networks are involved in many processes inside the cell and apart from cell–ECM and cell–cell contacts and also influence cell protrusion and motility (a), nucleus dynamics and genetic stability (b) as well as trafficking of organelles such as mitochondria and therefore cellular energetics (c). DSB, double-strand breaks.

It is becoming apparent that Arp2/3-dependent branched actin filaments lie at the cell–microenvironment interface and thus they establish a route for transmission of biological, chemical and mechanical signals from the surrounding environment to the cell core and vice versa (Figure 2). This allows a plethora of actions, from signalling, vesicle and organelle trafficking to gene expression and cellular energetics. Understanding the biomechanical roles of Arp2/3-dependent branched actin in higher levels of cellular organisation, from collective epithelia to the tissue and organism level, arises as an important challenge for the future.

## Author Contribution

VP and LMM drafted and edited the manuscript together. VP drew the Figures.

## Conflict of interest statement

Nothing declared.
